# A modified subgradient extragradient method for solving monotone variational inequalities

**DOI:** 10.1186/s13660-017-1366-3

**Published:** 2017-04-27

**Authors:** Songnian He, Tao Wu

**Affiliations:** 1 College of Science, Civil Aviation University of China, Tianjin, 300300 China; 2 Tianjin Key Laboratory for Advanced Signal Processing, Civil Aviation University of China, Tianjin, 300300 China

**Keywords:** 47J20, 90C25, 90C30, 90C52, variational inequalities, subgradient extragradient method, Lipschitz-continuous mapping, level set, half-spaces, convergence rate

## Abstract

In the setting of Hilbert space, a modified subgradient extragradient method is proposed for solving Lipschitz-continuous and monotone variational inequalities defined on a level set of a convex function. Our iterative process is relaxed and self-adaptive, that is, in each iteration, calculating two metric projections onto some half-spaces containing the domain is involved only and the step size can be selected in some adaptive ways. A weak convergence theorem for our algorithm is proved. We also prove that our method has $O(\frac{1}{n})$ convergence rate.

## Introduction

Let *H* be a real Hilbert space with inner product $\langle\cdot,\cdot \rangle$ and norm $\Vert \cdot \Vert $. The variational inequality problem (*VIP*) is aimed to finding a point $x^{*}\in C$, such that

1.1 $$ \bigl\langle f\bigl(x^{*}\bigr),x-x^{*} \bigr\rangle \geq0,\quad\forall x\in C, $$

where *C* is a nonempty closed convex subset of *H* and $f:C\rightarrow H$ is a given mapping. This problem and its solution set are denoted by $\operatorname{VI}(C,f)$ and $\operatorname {SOL}(C,f)$, respectively. We also always assume that $\operatorname{SOL}(C,f)\neq\emptyset$. The variational inequality problem $\operatorname{VI}(C,f)$ has received much attention due to its applications in a large variety of problems arising in structural analysis, economics, optimization, operations research and engineering sciences; see [1–14] and the references therein.

It is well known that the problem (1.1) is equivalent to the fixed point problem for finding a point $x^{*}\in C$, such that [1]

1.2 $$ x^{*}=P_{C}\bigl(x^{*}- \lambda f\bigl(x^{*}\bigr)\bigr), $$

where *λ* is an arbitrary positive constant. Many algorithms for the problem (1.1) are based on the fixed point problem (1.2). Korpelevich [2] proposed an algorithm for solving the problem (1.1) in Euclidean space $R^{n}$, known as the extragradient method (EG):

1.3 $$\begin{aligned}& x_{0}\in C, \\& \tilde{x}_{n}=P_{C} \bigl(x_{n}-\lambda f(x_{n})\bigr), \end{aligned}$$

1.4 $$\begin{aligned}& x_{n+1}=P_{C}\bigl(x_{n}-\lambda f( \tilde{x}_{n})\bigr), \end{aligned}$$

where *λ* is some positive number and $P_{C}$ denotes the metric projection of *H* onto *C*. She proved that if *f* is *κ*-Lipschitz-continuous and *λ* is selected such that $\lambda\in(0,1/\kappa)$, then the two sequences $\{ x_{n}\}$ and $\{\tilde{x}_{n}\}$ generated by the EG method, converge to the same point $z\in\operatorname{SOL}(C,f)$.

In 2006, Nadezhkina and Takahashi [3] generalized the above EG method to general Hilbert spaces (including infinite-dimensional spaces) and they also established the weak convergence theorem.

In each iteration of the EG method, in order to get the next iterate $x_{k+1}$, two projections onto *C* need to be calculated. But projections onto a general closed and convex subset are not easily executed and this might greatly affect the efficiency of the EG method. In order to overcome this weakness, Censor *et al.* developed the subgradient extragradient method in Euclidean space [4], in which the second projection in (1.4) onto *C* was replaced with a projection onto a specific constructible half-space, actually which is one of the subgradient half-spaces. Then, in [5, 6], Censor *et al.* studied the subgradient extragradient method for solving the *VIP* in Hilbert spaces. They also proved the weak convergence theorem under the assumption that *f* is a Lipschitzian continuous and monotone mapping.

The main purpose of this paper is to propose an improved subgradient extragradient method for solving the Lipschitz-continuous and monotone variational inequalities defined on a level set of a convex function [13], that is, $C:=\{x\in H \mid c(x)\leq0\}$ and $c:H\rightarrow R$ is a convex function. In our algorithm, two projections $P_{C}$ in (1.3) and (1.4) will be replaced with $P_{C_{k}}$ and $P_{T_{k}}$, respectively, where $C_{k}$ and $T_{k}$ are half-spaces, such that $C_{k}\supset C$ and $T_{k}\supset C$. $C_{k}$ is based on the subdifferential inequality, the idea of which was proposed firstly by Fukushima [14], and $T_{k}$ is the same one as Censor’s method [5].

It is also worth pointing out that the step size in our algorithm can be selected in some adaptive way, that is, we have no need to know or to estimate any information as regards the Lipschitz constant of *f*, therefore, our algorithm is easily executed.

Our paper is organized as follows. In Section 2, we list some basic definitions, properties and lemmas. In Section 3, the improved subgradient extragradient algorithm and its corresponding geometrical intuition are presented. In Section 4, the weak convergence theorem for our method is proved. Finally, we prove that our algorithm has $O(\frac {1}{n})$ convergence rate in the last section.

## Preliminaries

In this section, we list some basic concepts and lemmas, which are useful for constructing the algorithm and analyzing the convergence. Let *H* be a real Hilbert space with inner product $\langle\cdot,\cdot \rangle$ and norm $\Vert \cdot \Vert $ and let *C* be a closed convex subset of *H*. We write $x_{k}\rightharpoonup x$ and $x_{k}\rightarrow x$ to indicate that the sequence $\{x_{k}\}_{k=0}^{\infty}$ converges weakly and strongly to *x*, respectively. For each point $x\in H$, there exists a unique nearest point in *C*, denoted by $P_{C}(x)$, such that

2.1 $$ \bigl\Vert x-P_{C}(x) \bigr\Vert \leq \Vert x-y \Vert ,\quad \forall y\in C. $$

The mapping $P_{C}:H\rightarrow C$ is called the metric projection of *H* onto *C*. It is well known that $P_{C}$ is characterized by the following inequalities:

2.2 $$\begin{aligned}& \bigl\langle x-P_{C}(x),P_{C}(x)-y\bigr\rangle \geq0, \end{aligned}$$

2.3 $$\begin{aligned}& \Vert x-y \Vert ^{2}\geq \bigl\Vert x-P_{C}(x) \bigr\Vert ^{2}+ \bigl\Vert y-P_{C}(x) \bigr\Vert ^{2}, \end{aligned}$$

for all $x\in H$, $y\in C$ [15, 16].

A function $c:H\rightarrow R$ is said to be Gâteaux differentiable at $x\in H$, if there exists an element, denoted by $c'(x)\in H$, such that

$$\lim_{t\rightarrow0}\frac{c(x+tv)-c(x)}{t}=\bigl\langle v,c'(x) \bigr\rangle ,\quad \forall v\in H, $$

where $c'(x)$ is called the Gâteaux differential of *c* at *x*. We say *c* is Gâteaux differentiable on *H*, if for each $x\in H$, *c* is Gâteaux differentiable at *x*.

A function $c:H\rightarrow R$ is said to be weakly lower semicontinuous (w-lsc) at $x\in H$, if $x_{k}\rightharpoonup x$ implies $c(x)\leq\liminf_{k\rightarrow\infty}c(x_{k})$. We say *c* is weakly lower semicontinuous on *H*, if for each $x\in H$, *c* is weakly lower semicontinuous at *x*.

A function $c:H\rightarrow R$ is called convex, if we have the inequality

$$c\bigl(tx+(1-t)y\bigr)\leq tc(x)+(1-t)c(y), $$

for all $t\in[0,1]$ and $x,y\in H$.

For a convex function $c:H\rightarrow R$, *c* is said to be subdifferentiable at a point $x\in H$ if the set

2.4 $$\begin{aligned} \partial c(x)\triangleq\bigl\{ d\in H \mid c(y)\geq c(x)+\langle d,y-x\rangle ,\forall y\in H\bigr\} \end{aligned}$$

is not empty, where each element in $\partial c(x)$ is called a subgradient of *c* at *x*, $\partial c(x)$ is subdifferential of *c* at *x* and the inequality in (2.4) is said to be the subdifferential inequality of *c* at *x*. We say *c* is subdifferentiable on *H*, if *c* is subdifferentiable at each $x\in H$. It is well known that if *c* is Gâteaux differentiable at *x*, then *c* is subdifferentiable at *x* and $\partial c(x)=\{c'(x)\}$, namely, $\partial c(x)$ is just a set of the simple points [17].

A mapping $f:H \rightarrow H$ is said to be Lipschitz-continuous [15], if there exists a positive constant *κ*, such that

$$\bigl\Vert f(x)-f(y) \bigr\Vert \leq\kappa \Vert x-y \Vert ,\quad\forall x,y \in H. $$

*f* is also said to be a *κ*-Lipschitzian-continuous mapping.

A mapping $f:H \rightarrow H$ is said to be monotone on *H*, if

$$\bigl\langle f(x)-f(y),x-y\bigr\rangle \geq0,\quad\forall x,y\in H. $$

### Definition 2.1

Normal cone

We denote the normal cone by $N_{C}(v)$ [18] of *C* at $v\in C$, *i.e.*

2.5 $$ N_{C}(v):=\bigl\{ w\in H \mid\langle w,y-v\rangle\leq0 ,\forall y\in C\bigr\} . $$

### Definition 2.2

Maximal monotone operator

Let $T:H\rightrightarrows2^{H}$ be a point-to-set operator defined on *H*. *T* is called a maximal monotone operator if *T* is monotone, *i.e.*

$$ \langle u-v,x-y\rangle\geq0 ,\quad\forall u\in T(x)\mbox{ and } \forall v\in T(y) $$

and the graph $G(T)$ of *T*,

$$ G(T):=\bigl\{ (x,u)\in H\times H \mid u \in T(x)\bigr\} , $$

is not properly contained in the graph of any other monotone operator.

It is clear that a monotone mapping *T* is maximal iff for any $(x,u)\in H\times H$, if $\langle u-v,x-y\rangle\geq0$, $\forall(y,v)\in G(T)$, then it follows that $u\in T(x)$.

Define

$$ Tv= \textstyle\begin{cases}f(v)+N_{C}(v)&\mbox{if }v\in C,\\ \emptyset,&\mbox{if } v\notin C. \end{cases} $$

Then *T* is maximal monotone and $0\in Tv$ if and only if $v\in \operatorname{SOL}(C,f)$ [18].

The next property is known as the Opial condition and all Hilbert spaces have this property [19].

### Lemma 2.3

*For any sequence* $\{x_{k}\}_{k=0}^{\infty}$ *in* *H* *that converges weakly to* *x* ($x_{k}\rightharpoonup x$), *the inequality*

2.6 $$ \liminf_{n\rightarrow\infty} \Vert x_{k}-x \Vert < \liminf_{n\rightarrow\infty} \Vert x_{k}-y \Vert $$

*holds for any* $y\in H$ *with* $x\neq y$.

The following lemma was proved in [20].

### Lemma 2.4

*Let* *H* *be a real Hilbert space and let* *C* *be a nonempty*, *closed and convex subset of* *H*. *Let the sequence* $\{x_{k}\}_{k=0}^{\infty}\subset H$ *be Fejér*-*monotone with respect to* *C*, *i*.*e*., *for any* $u\in C$,

2.7 $$ \Vert x_{k+1}-u \Vert \leq \Vert x_{k}-u \Vert ,\quad\forall k\geq0. $$

*Then* $\{P_{C}(x_{k})\}_{k=0}^{\infty}$ *converges strongly to some* $z'\in C$.

## The modified subgradient extragradient method

In this section, we give our algorithm for solving the $\operatorname {VI}(C,f)$ in the setting of Hilbert spaces, where *C* is a level set given as follows:

3.1 $$ C:=\bigl\{ x\in H \mid c(x)\leq0\bigr\} , $$

where $c:H\rightarrow R$ is a convex function.

In the rest of this paper, we always assume that the following conditions are satisfied.

### Condition 3.1

The solution set of $\operatorname{VI}(C,f)$, denoted by $\operatorname {SOL}(C,f)$, is nonempty.

### Condition 3.2

The mapping $f:H\rightarrow H$ is monotone and Lipschitz-continuous on *H* (but we have no need to know or to estimate the Lipschitz constant of *f*).

### Condition 3.3

The function $c:H\rightarrow R$ satisfies the following conditions:

(i) $c(x)$ is a convex function;
(ii) $c(x)$ is weakly lower semicontinuous on *H*;
(iii) $c(x)$ is Gâteaux differentiable on *H* and $c'(x)$ is a $M_{1}$-Lipschitzian-continuous mapping on *H*;
(iv) there exists a positive constant $M_{2}$ such that $\Vert f(x) \Vert \leq M_{2} \Vert c'(x) \Vert $ for any $x\in\partial C$, where *∂C* denotes the boundary of *C*.

Next, we present the modified subgradient extragradient method as follows.

### Algorithm 3.4

The modified subgradient extragradient method

Step 1: select an initial guess $x_{0}\in H$ arbitrarily, set $k=0$ and construct the half-space
  $$ C_{k}:=\bigl\{ w\in H \mid c(x_{k})+\bigl\langle c'(x_{k}),w-x_{k}\bigr\rangle \leq0\bigr\} ; $$
Step 2: given the current iteration $x_{k}$, compute
  3.2 $$ y_{k}=P_{C_{k}}\bigl(x_{k}- \beta_{k}f(x_{k})\bigr), $$
  where
  3.3 $$\begin{aligned} \beta_{k}=\sigma\rho^{m_{k}},\quad\sigma>0, \rho\in(0,1) \end{aligned}$$
  and $m_{k}$ is the smallest nonnegative integer, such that
  3.4 $$\begin{aligned} \beta_{k}^{2} \bigl\Vert f(x_{k})-f(y_{k}) \bigr\Vert ^{2}+2M \beta_{k} \Vert x_{k}-y_{k} \Vert ^{2}\leq\nu^{2} \Vert x_{k}-y_{k} \Vert ^{2}, \end{aligned}$$
  where $M=M_{1}M_{2}$ and $\nu\in(0,1)$.
Step 3: calculate the next iterate,
  3.5 $$ x_{k+1}=P_{T_{k}}\bigl(x_{k}- \beta_{k}f(y_{k})\bigr), $$
  where
  3.6 $$ T_{k}=\bigl\{ w\in H \mid\bigl\langle x_{k}- \beta _{k}f(x_{k})-y_{k},w-y_{k}\bigr\rangle \leq0\bigr\} , $$
  which is the same half-space as Censor’s method [5].

**Figure 1:**
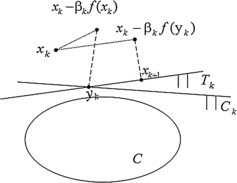
$\pmb{x_{k+1}}$ **is the subgradient extragradient projection of point** $\pmb{x_{k}-\beta_{k}f(y_{k})}$ **onto the hyperplane** $\pmb{T_{k}}$ **.**

Figure 1 illustrates the iterative steps of this algorithm.

At the end of this section, we list the alternating theorem [21, 22] for the solutions of $\operatorname{VI}(C,f)$, where *C* is given by (3.1). This result will be used to prove the convergence theorem of our algorithm in the next section.

### Theorem 3.5

*Assume that the solution set* $\operatorname{SOL}(C,f)$ *of* $\operatorname {VI}(C,f)$ *is nonempty*. *Given* $x^{*}\in C$. *Then* $x^{*}\in\operatorname{SOL}(C,f)$ *iff we have either*

1. $f(x^{*})=0$, *or*
2. $x^{*}\in\partial C$ *and there exists a positive constant* *β* *such that* $f(x^{*})=-\beta c'(x^{*})$.

## Convergence theorem of the algorithm

In this section, we prove the weak convergence theorem for Algorithm 3.4. First of all, we give the following lemma, which plays a crucial role in the proof of our main result.

### Lemma 4.1

*Let* $\{x_{k}\}_{k=0}^{\infty}$ *and* $\{y_{k}\}_{k=0}^{\infty}$ *be the two sequences generated by Algorithm* 3.4. *Let* $u\in\operatorname{SOL}(C,f)$ *and let* $\beta_{k}$ *be selected as* (3.3) *and* (3.4). *Then*, *under the Conditions* 3.1, 3.2 *and* 3.3, *we have*

4.1 $$ \Vert x_{k+1}-u \Vert ^{2}\leq \Vert x_{k}-u \Vert ^{2}-\bigl(1-\nu^{2}\bigr) \Vert x_{k}-y_{k} \Vert ^{2},\quad\forall k\geq0. $$

### Proof

Taking $u\in\operatorname{SOL}(C,f)$ arbitrarily, for all $k\geq0$, using (2.3) and the monotonicity of *f*, we have

4.2 $$\begin{aligned} \Vert x_{k+1}-u \Vert ^{2}&\leq \bigl\Vert x_{k}-\beta _{k}f(y_{k})-u \bigr\Vert ^{2}- \bigl\Vert x_{k}-\beta _{k}f(y_{k})-x_{k+1} \bigr\Vert ^{2} \\ &= \Vert x_{k}-u \Vert ^{2}- \Vert x_{k}-x_{k+1} \Vert ^{2}+2\beta_{k} \bigl\langle f(y_{k}),u-x_{k+1}\bigr\rangle \\ &= \Vert x_{k}-u \Vert ^{2}- \Vert x_{k}-x_{k+1} \Vert ^{2}+2\beta_{k} \bigl[\bigl\langle f(y_{k})-f(u),u-y_{k}\bigr\rangle \\ &\quad{}+\bigl\langle f(u),u-y_{k}\bigr\rangle +\bigl\langle f(y_{k}),y_{k}-x_{k+1}\bigr\rangle \bigr] \\ &\leq \Vert x_{k}-u \Vert ^{2}- \Vert x_{k}-x_{k+1} \Vert ^{2} \\ &\quad{}+2\beta_{k}\bigl[\bigl\langle f(u),u-y_{k}\bigr\rangle + \bigl\langle f(y_{k}),y_{k}-x_{k+1}\bigr\rangle \bigr] \\ &= \Vert x_{k}-u \Vert ^{2}- \Vert x_{k}-y_{k} \Vert ^{2}- \Vert y_{k}-x_{k+1} \Vert ^{2}-2\langle x_{k}-y_{k},y_{k}-x_{k+1}\rangle \\ &\quad{}+2\beta_{k}\bigl[\bigl\langle f(u),u-y_{k}\bigr\rangle + \bigl\langle f(y_{k}),y_{k}-x_{k+1}\bigr\rangle \bigr] \\ &= \Vert x_{k}-u \Vert ^{2}- \Vert x_{k}-y_{k} \Vert ^{2}- \Vert y_{k}-x_{k+1} \Vert ^{2} \\ &\quad{}+2\bigl\langle x_{k}-\beta_{k}f(y_{k})-y_{k},x_{k+1}-y_{k} \bigr\rangle +2\beta _{k}\bigl\langle f(u),u-y_{k}\bigr\rangle . \end{aligned}$$

By the definition of $T_{k}$, we get

4.3 $$ \begin{aligned}[b] &\bigl\langle x_{k}- \beta_{k}f(y_{k})-y_{k},x_{k+1}-y_{k} \bigr\rangle \\ &\quad =\bigl\langle x_{k}-\beta_{k}f(x_{k})-y_{k},x_{k+1}-y_{k} \bigr\rangle +\beta_{k}\bigl\langle f(x_{k})- f(y_{k}),x_{k+1}-y_{k}\bigr\rangle \\ &\quad \leq \beta_{k}\bigl\langle f(x_{k})- f(y_{k}),x_{k+1}-y_{k} \bigr\rangle \\ &\quad \leq \beta_{k} \bigl\Vert f(x_{k})- f(y_{k}) \bigr\Vert \Vert x_{k+1}-y_{k} \Vert . \end{aligned} $$

Substituting (4.3) into the last inequality of (4.2), thus we obtain

4.4 $$ \begin{aligned}[b] \Vert x_{k+1}-u \Vert ^{2}&\leq \Vert x_{k}-u \Vert ^{2}- \Vert x_{k}-y_{k} \Vert ^{2}- \Vert y_{k}-x_{k+1} \Vert ^{2} \\ &\quad{}+2\beta_{k} \bigl\Vert f(x_{k})-f(y_{k}) \bigr\Vert \Vert x_{k+1}-y_{k} \Vert \\ &\quad{}+2\beta_{k}\bigl\langle f(u),u-y_{k}\bigr\rangle \\ &\leq \Vert x_{k}-u \Vert ^{2}- \Vert x_{k}-y_{k} \Vert ^{2}+2\beta_{k} \bigl\langle f(u),u-y_{k}\bigr\rangle \\ &\quad{}+\beta_{k}^{2} \bigl\Vert f(x_{k})-f(y_{k}) \bigr\Vert ^{2}. \end{aligned} $$

The subsequent proof is divided into following two cases.

Case 1: $f(u)\neq0$.

Using Theorem 3.5, there exists a $\beta_{u}> 0$ such that $f(u)=-\beta _{u}c'(u)$. By the subdifferential inequality, we have

4.5 $$ c(u)+\bigl\langle c'(u),y_{k}-u\bigr\rangle \leq c(y_{k}),\quad\forall k\geq0. $$

Noting the fact that $c(u)=0$ due to $u\in\partial C$, we have

4.6 $$ \bigl\langle c'(u),y_{k}-u\bigr\rangle \leq c(y_{k}),\quad\forall k\geq0. $$

Since $-\beta_{u}<0$, it follows from (4.6) that

$$\bigl\langle -\beta_{u}c'(u),y_{k}-u\bigr\rangle \geq- \beta_{u}c(y_{k}),\quad \forall k\geq0, $$

it implies

$$\bigl\langle f(u),y_{k}-u\bigr\rangle \geq- \beta_{u}c(y_{k}), \quad\forall k\geq0 $$

or

4.7 $$ \bigl\langle f(u), u-y_{k}\bigr\rangle \leq \beta_{u}c(y_{k}),\quad\forall k\geq0. $$

By the definition of $C_{k}$, we have

$$c(x_{k})+\bigl\langle c'(x_{k}), y_{k}-x_{k}\bigr\rangle \leq0,\quad\forall k\geq0, $$

using the subdifferential inequality again,

$$c(y_{k})+\bigl\langle c'(y_{k}), x_{k}-y_{k}\bigr\rangle \leq c(x_{k}),\quad \forall k\geq0. $$

Adding the above two inequalities, we obtain

4.8 $$ c(y_{k})\leq\bigl\langle c'(y_{k})-c'(x_{k}), y_{k}-x_{k}\bigr\rangle ,\quad \forall k\geq0. $$

Combining (4.7) and (4.8), we have by using (iii) and (iv) of Condition 3.3

4.9 $$ \begin{aligned}[b] \bigl\langle f(u), u-y_{k} \bigr\rangle &\leq \beta_{u}c(y_{k}) \\ &\leq \beta_{u}\bigl\langle c'(y_{k})-c'(x_{k}), y_{k}-x_{k}\bigr\rangle \\ &\leq M \Vert y_{k}-x_{k} \Vert ^{2}, \end{aligned} $$

where *M* is defined as before. Substituting (4.9) into the last inequality of (4.4), we obtain

$$ \begin{aligned} \Vert x_{k+1}-u \Vert ^{2}& \leq \Vert x_{k}-u \Vert ^{2}- \Vert x_{k}-y_{k} \Vert ^{2}+2M\beta_{k} \Vert y_{k}-x_{k} \Vert ^{2} \\ &\quad{}+\beta_{k}^{2} \bigl\Vert f(x_{k})-f(y_{k}) \bigr\Vert ^{2}. \end{aligned} $$

Finally, from the condition of $\beta_{k}$ given by (3.4), we get

$$ \Vert x_{k+1}-u \Vert ^{2}\leq \Vert x_{k}-u \Vert ^{2}-\bigl(1-\nu^{2}\bigr) \Vert x_{k}-y_{k} \Vert ^{2}. $$

Case 2: $f(u)=0$.

From (4.4), we can easily get

4.10 $$ \begin{aligned} \Vert x_{k+1}-u \Vert ^{2}&\leq \Vert x_{k}-u \Vert ^{2}- \Vert x_{k}-y_{k} \Vert ^{2}+\beta_{k}^{2} \bigl\Vert f(x_{k})-f(y_{k}) \bigr\Vert ^{2}. \end{aligned} $$

Obviously, (3.4) implies

4.11 $$\begin{aligned} \beta_{k}^{2} \bigl\Vert f(x_{k})-f(y_{k}) \bigr\Vert ^{2}\leq \nu^{2} \Vert x_{k}-y_{k} \Vert ^{2},\quad\nu\in(0,1). \end{aligned}$$

Thus, (4.1) follows from the combination of (4.10) and (4.11). □

Indeed, substituting (3.3) into (3.4), we get

4.12 $$ \bigl(\sigma\rho^{m_{k}}\bigr)^{2} \bigl\Vert f(x_{k})-f(y_{k}) \bigr\Vert ^{2}+2M\sigma \rho^{m_{k}} \Vert x_{k}-y_{k} \Vert ^{2}\leq\nu ^{2} \Vert x_{k}-y_{k} \Vert ^{2}. $$

Let *m* be the smallest nonnegative integer, such that

4.13 $$\begin{aligned} \bigl(\sigma\rho^{m}\bigr)^{2} \kappa^{2}+2M\sigma\rho^{m}\leq\nu^{2} , \end{aligned}$$

where *κ* is the Lipschitz constant of *f*. Noting that $\Vert f(x_{k})-f(y_{k}) \Vert \leq\kappa \Vert x_{k}-y_{k} \Vert $, we assert from (4.12) and (4.13) that $m_{k}\leq m$, which implies

4.14 $$ \beta_{k}\geq\sigma\rho^{m}, $$

namely $\inf_{k\geq0}\{\beta_{k}\}>0$.

### Theorem 4.2

*Assume that Conditions* 3.1-3.3 *hold*. *Then the two sequences* $\{x_{k}\} _{k=0}^{\infty}$ *and* $\{y_{k}\}_{k=0}^{\infty}$ *generated by Algorithm* 3.4 *converge weakly to the same point* $z \in\operatorname{SOL}(C,f)$, *furthermore*

4.15 $$ z=\lim_{k\rightarrow\infty}P_{\operatorname{SOL}(C,f)}(x_{k}). $$

### Proof

By Lemma 4.1,

$$\Vert x_{k+1}-u \Vert ^{2}\leq \Vert x_{k}-u \Vert ^{2} $$

for all $k\geq0$, so there exists

$$a=\lim_{k\rightarrow\infty} \Vert x_{k}-u \Vert $$

and the sequence $\{x_{k}\}_{k=0}^{\infty}$ is bounded. From (4.1), we have

$$\Vert x_{k}-y_{k} \Vert ^{2}\leq \frac{1}{1-\nu^{2}}\bigl[ \Vert x_{k}-u \Vert ^{2}- \Vert x_{k+1}-u \Vert ^{2}\bigr]. $$

Hence,

4.16 $$ x_{k}-y_{k}\rightarrow0\quad(k\rightarrow \infty). $$

In addition,

$$f(x_{k})- f(y_{k})\rightarrow0\quad(k\rightarrow\infty). $$

Using the Cauchy-Schwartz inequality,

$$ \Vert y_{k} \Vert = \Vert y_{k}-x_{k}+x_{k} \Vert \leq \Vert y_{k}-x_{k} \Vert + \Vert x_{k} \Vert , $$

hence, the sequence $\{y_{k}\}_{k=0}^{\infty}$ is also bounded.

Let $\omega(x_{k})$ be the set of weak limit points of $\{x_{k}\} _{k=0}^{\infty}$, *i.e.*,

$$\omega(x_{k})=\bigl\{ z \mid\exists \{x_{k_{j}} \}_{j=0}^{\infty}\subset \{x_{k}\}_{k=0}^{\infty} \mbox{ s.t. } x_{k_{j}}\rightharpoonup z\bigr\} . $$

Since the sequence $\{x_{k}\}_{k=0}^{\infty}$ is bounded, $\omega (x_{k})\neq\emptyset$. Taking $z\in\omega(x_{k})$ arbitrarily, there exists some subsequence $\{x_{k_{j}}\}_{j=0}^{\infty}$ of $\{x_{k}\}_{k=0}^{\infty}$, such that

4.17 $$ x_{k_{j}}\rightharpoonup z\quad(j\rightarrow\infty). $$

Equation (4.17) together with (4.16) leads to

4.18 $$ y_{k_{j}}\rightharpoonup z\quad(j\rightarrow\infty). $$

Due to $y_{k}\in C_{k}$ and the definition of $C_{k}$, we have

4.19 $$ c(x_{k})-\bigl\langle c'(x_{k}),x_{k}-y_{k} \bigr\rangle \leq0, $$

then, using the Cauchy-Schwartz inequality again,

4.20 $$ \begin{aligned} c(x_{k})&\leq \bigl\Vert c'(x_{k}) \bigr\Vert \Vert x_{k}-y_{k} \Vert . \end{aligned} $$

According to (iii) in Condition 3.3, we can deduce that $c'(x)$ is bounded on any bounded sets of *H*, so there exists $M'>0$ such that $\Vert c'(x_{k}) \Vert \leq M'$ for all $k\geq0$, and then

$$ c(x_{k})\leq M' \Vert x_{k}-y_{k} \Vert \rightarrow0 \quad (k\rightarrow\infty). $$

According to (ii) in Condition 3.3, we have

$$c(z)\leq\liminf_{j\rightarrow\infty} c(x_{k_{j}})\leq0. $$

Hence, $z\in C$.

Now, we turn to showing $z\in\operatorname{SOL}(C,f)$. Define

$$ Tv= \textstyle\begin{cases}f(v)+N_{C}(v), &\mbox{if } v\in C,\\ \emptyset,&\mbox{if } v\notin C, \end{cases} $$

where $N_{C}(v)$ is defined by (2.5). Obviously, *T* is a maximal monotone operator.

For arbitrary $(v,w)\in G(T)$, we have

$$w\in T(v)=f(v)+N_{C}(v), $$

equivalently,

$$w-f(v)\in N_{C}(v). $$

Setting $y=z$ in (2.5), we get

4.21 $$ \bigl\langle w-f(v),z-v \bigr\rangle \leq0. $$

On the other hand, by the definition of $y_{k}$ and (2.2), we have

$$ \bigl\langle x_{k}-\beta_{k}f(x_{k})-y_{k},y_{k}-v \bigr\rangle \geq0 $$

or

4.22 $$ \biggl\langle \frac{y_{k}-x_{k}}{\beta_{k}}+f(x_{k}),v-y_{k} \biggr\rangle \geq0, $$

for all $k\geq0$. Using (4.21) and (4.22), we obtain

4.23 $$ \begin{aligned}[b] \langle w, v-z\rangle&\geq\bigl\langle f(v) , v-z\bigr\rangle \\ &\geq\bigl\langle f(v) , v-z\bigr\rangle -\biggl\langle \frac{y_{k_{j}}-x_{k_{j}}}{\beta _{k_{j}}}+f(x_{k_{j}}),v-y_{k_{j}} \biggr\rangle \\ &=\bigl\langle f(v) , v-y_{k_{j}}+y_{k_{j}}-z\bigr\rangle -\biggl\langle \frac {y_{k_{j}}-x_{k_{j}}}{\beta_{k_{j}}}+f(x_{k_{j}}),v-y_{k_{j}}\biggr\rangle \\ &=\bigl\langle f(v) , v-y_{k_{j}}\bigr\rangle +\bigl\langle f(v) , y_{k_{j}}-z\bigr\rangle \\ &\quad {}-\biggl\langle \frac{y_{k_{j}}-x_{k_{j}}}{\beta _{k_{j}}}+f(x_{k_{j}}),v-y_{k_{j}}\biggr\rangle \\ &=\bigl\langle f(v)-f(y_{k_{j}}) , v-y_{k_{j}}\bigr\rangle +\bigl\langle f(y_{k_{j}})-f(x_{k_{j}}) , v-y_{k_{j}}\bigr\rangle \\ &\quad {}-\biggl\langle \frac{y_{k_{j}}-x_{k_{j}}}{\beta_{k_{j}}},v-y_{k_{j}}\biggr\rangle +\bigl\langle f(v) , y_{k_{j}}-z\bigr\rangle \\ &\geq\bigl\langle f(v) , y_{k_{j}}-z\bigr\rangle +\bigl\langle f(y_{k_{j}})-f(x_{k_{j}}) , v-y_{k_{j}}\bigr\rangle \\ &\quad {}-\biggl\langle \frac{y_{k_{j}}-x_{k_{j}}}{\beta_{k_{j}}},v-y_{k_{j}}\biggr\rangle . \end{aligned} $$

By virtue of (4.14), (4.16) and Condition 3.2, taking $j\rightarrow \infty$ in (4.23), we have

4.24 $$ \langle w,v-z \rangle\geq0. $$

Since *T* is a maximal monotone operator, (4.24) means that $0\in T(z)$ and consequently $z\in T^{-1}(0)=\operatorname{SOL}(C,f)$.

Now we are in a position to verify that $x_{k}\rightharpoonup z$ ($k\rightarrow\infty$). In fact, if there exists another subsequence $\{x_{k_{i}}\} _{i=0}^{\infty}$ of $\{x_{k}\}_{k=0}^{\infty}$, such that $x_{k_{i}}\rightharpoonup\bar{z}\in\operatorname {SOL}(C,f)$, but $\bar{z}\neq z$, noting the fact that $\{ \Vert x_{k}-u \Vert \}_{k=0}^{\infty}$ is decreasing for all $u\in\operatorname{SOL}(C,f)$, we obtain by using Lemma 2.3

4.25 $$ \begin{aligned}[b] \lim_{k\rightarrow\infty} \Vert x_{k}-z \Vert &=\lim_{j\rightarrow\infty} \Vert x_{k_{j}}-z \Vert < \lim_{j\rightarrow\infty} \Vert x_{k_{j}}-\bar{z} \Vert \\ &=\lim_{k\rightarrow\infty} \Vert x_{k}-\bar{z} \Vert =\lim _{i\rightarrow\infty} \Vert x_{k_{i}}-\bar{z} \Vert \\ &< \lim_{i\rightarrow\infty} \Vert x_{k_{i}}-z \Vert =\lim _{k\rightarrow\infty} \Vert x_{k}-z \Vert . \end{aligned} $$

This is a contradiction, so $\bar{z}=z$. Consequently, we have $x_{k}\rightharpoonup z$ ($k\rightarrow\infty$) and $y_{k}\rightharpoonup z$ ($k\rightarrow\infty$).

Finally, we show that $z=\lim_{k\rightarrow\infty}P_{\operatorname {SOL}(C,f)}(x_{k})$. Put $u_{k}=P_{\operatorname{SOL}(C,f)}(x_{k})$, using (2.2) again and $z\in\operatorname{SOL}(C,f)$,

4.26 $$ \langle x_{k}-u_{k},u_{k}-z \rangle\geq0. $$

By Lemma 2.4, there exists $u^{*}\in\operatorname{SOL}(C,f)$ such that $u_{k}\rightarrow u^{*}$. Therefore, taking $k\rightarrow\infty$ in (4.26), we have

4.27 $$ \bigl\langle z-u^{*},u^{*}-z \bigr\rangle \geq0, $$

therefore $z=u^{*}$. The proof is complete. □

## Convergence rate of the modified method

In this section, we prove the convergence rate of our modified subgradient extragradient method in the ergodic sense. The base of the complexity proof is ([23, 24])

5.1 $$\begin{aligned} \operatorname{SOL}(C,f)=\bigcap_{u\in C} \bigl\{ z\in C \mid\bigl\langle f(u),u-z\bigr\rangle \geq0\bigr\} . \end{aligned}$$

In order to prove the convergence rate, now we give the key inequality of our method. Indeed, by an argument very similar to the proof of Lemma 4.1, it is not difficult to get the following result.

### Lemma 5.1

*Let* $\{x_{k}\}_{k=0}^{\infty}$ *and* $\{y_{k}\}_{k=0}^{\infty}$ *be the two sequences generated by Algorithm* 3.4 *and let* $\beta_{k}$ *be selected as* (3.3) *and* (3.4). *Assume that the Conditions* 3.1, 3.2 *and* 3.3 *are satisfied*. *Then*, *for any* $u\in C$, *we have*

5.2 $$ \Vert x_{k+1}-u \Vert ^{2}\leq \Vert x_{k}-u \Vert ^{2}-\bigl(1-\nu^{2}\bigr) \Vert x_{k}-y_{k} \Vert ^{2}+2\beta_{k} \bigl\langle f(u), u-y_{k} \bigr\rangle . $$

### Theorem 5.2

*For any integer* $n>0$, *we have a* $z_{n}\in H$, *which satisfies* $z_{n}\rightharpoonup z$, $z\in\operatorname{SOL}(C,f)$ *and*

5.3 $$ \bigl\langle f(u), z_{n}-u \bigr\rangle \leq \frac{ \Vert x_{0}-u \Vert ^{2}}{\Upsilon_{n}},\quad\forall u\in C, $$

*where*

5.4 $$ z_{n}=\frac{\sum^{n}_{k=0}2\beta_{k}y_{k}}{\Upsilon_{n}} \quad\textit {and}\quad \Upsilon_{n}=\sum^{n}_{k=0}2 \beta_{k}. $$

### Proof

Using (5.2), we get

5.5 $$ 2\beta_{k}\bigl\langle f(u), y_{k}-u \bigr\rangle \leq \Vert x_{k}-u \Vert ^{2}- \Vert x_{k+1}-u \Vert ^{2}. $$

Summing the inequality (5.5) over $k=0,1,\ldots,n$, we get

$$\Biggl\langle f(u) , \sum^{n}_{k=0}2 \beta_{k}y_{k}-\sum^{n}_{k=0}2 \beta_{k}u \Biggr\rangle \leq \Vert x_{0}-u \Vert ^{2},\quad\forall u\in C. $$

From the notation of $\Upsilon_{n}$ and $z_{n}$ in (5.4), we derive

$$ \bigl\langle f(u), z_{n}-u \bigr\rangle \leq\frac{ \Vert x_{0}-u \Vert ^{2}}{\Upsilon_{n}},\quad \forall u\in C. $$

On the other hand, since $z_{n}$ is a convex combination of $y_{0}, y_{1}, \ldots, y_{n}$, it is easy to see that $z_{n}\rightharpoonup z\in\operatorname {SOL}(C,f)$ due to the fact that $y_{k}\rightharpoonup z\in\operatorname{SOL}(C,f)$ proved by Theorem 4.2. The proof is complete. □

Let $\beta=\sigma\rho^{m}$. From (4.14), $\beta_{k}\geq\beta$ holds for all $k\geq0$ and this together with (5.4) leads to

$$\Upsilon_{n}\geq2(n+1)\beta, $$

this means the modified subgradient extragradient method has $O(\frac {1}{n})$ convergence rate. In fact, for any bounded subset $D\subset C$ and given accuracy $\epsilon>0$, our algorithm achieves

$$\bigl\langle f(u), z_{n}-u \bigr\rangle \leq\epsilon,\quad\forall u\in D, $$

in at most

$$n=\biggl\lceil \frac{m}{2\beta\epsilon}\biggr\rceil $$

iterations, where $z_{n}$ defined in (5.4) and $m=\sup\{ \Vert x_{0}-u \Vert ^{2} \mid u\in D\}$.

## Results and discussion

Since the modified subgradient extragradient method proposed in this paper is relaxed and self-adaptive, it is easily implemented. A weak convergence theorem for our algorithm is proved due to the alternating theorem for the solutions of variational inequalities. Our results in this paper effectively improve the existing related results.

## Conclusion

Although the extragradient methods and the subgradient extragradient methods have been widely studied, the existing algorithms all face the problem that the projection operator is hard to calculate. The problem can be solved effectively by using the modified subgradient extragradient method proposed in this paper, since two projections onto the original domain are all replaced with projections onto some half-spaces, which is very easily calculated. Besides, the step size can be selected in some adaptive ways, which means that we have no need to know or to estimate the Lipschitz constant of the operator. Furthermore, we prove that our method has $O(\frac{1}{n})$ convergence rate.
